# Internet of Vehicles (IoV)-Based Task Scheduling Approach Using Fuzzy Logic Technique in Fog Computing Enables Vehicular Ad Hoc Network (VANET)

**DOI:** 10.3390/s24030874

**Published:** 2024-01-29

**Authors:** Muhammad Ehtisham, Mahmood ul Hassan, Amin A. Al-Awady, Abid Ali, Muhammad Junaid, Jahangir Khan, Yahya Ali Abdelrahman Ali, Muhammad Akram

**Affiliations:** 1 Department of IT, The University of Haripur, Haripur 22620, Pakistan; muhammadehti97@gmail.com (M.E.); mjunaid@uoh.edu.pk (M.J.); 2 Department of Computer Skills, Deanship of Preparatory Year, Najran University, Najran 66241, Saudi Arabia; aaalawady@nu.edu.sa; 3 Department of Computer Science, University of Engineering and Technology, Taxila 47050, Pakistan; 4 Department of Computer Science, GANK(S) DC KTS, Haripur 22620, Pakistan; 5 Department of Computer Science, Applied College Mohyail Asir, King Khalid University, Abha 62529, Saudi Arabia; jhkhan@kku.edu.sa; 6 Department of Information Systems, Faculty Computer Science and Information System, Najran University, Najran 66241, Saudi Arabia; yaali@nu.edu.sa; 7 Department of Computer Science, College of Computer Science and Information Systems, Najran University, Najran 66241, Saudi Arabia; akram.moghal@gmail.com

**Keywords:** task scheduling, vehicular ad hoc network, fuzzy logic, fog computing

## Abstract

The intelligent transportation system (ITS) relies heavily on the vehicular ad hoc network (VANET) and the internet of vehicles (IoVs), which combine cloud and fog to improve task processing capabilities. As a cloud extension, the fog processes’ infrastructure is close to VANET, fostering an environment favorable to smart cars with IT equipment and effective task management oversight. Vehicle processing power, bandwidth, time, and high-speed mobility are all limited in VANET. It is critical to satisfy the vehicles’ requirements for minimal latency and fast reaction times while offloading duties to the fog layer. We proposed a fuzzy logic-based task scheduling system in VANET to minimize latency and improve the enhanced response time when offloading tasks in the IoV. The proposed method effectively transfers workloads to the fog computing layer while considering the constrained resources of car nodes. After choosing a suitable processing unit, the algorithm sends the job and its associated resources to the fog layer. The dataset is related to crisp values for fog computing for system utilization, latency, and task deadline time for over 5000 values. The task execution, latency, deadline of task, storage, CPU, and bandwidth utilizations are used for fuzzy set values. We proved the effectiveness of our proposed task scheduling framework via simulation tests, outperforming current algorithms in terms of task ratio by 13%, decreasing average turnaround time by 9%, minimizing makespan time by 15%, and effectively overcoming average latency time within the network parameters. The proposed technique shows better results and responses than previous techniques by scheduling the tasks toward fog layers with less response time and minimizing the overall time from task submission to completion.

## 1. Introduction

In VANET, the task handling and scheduling required is one of the most challenging tasks due to its mobility behavior. Task scheduling in VANET is now one of the most effective ways to handle high computational and energy-consuming tasks. The roadside unit (RSU) computes tasks’ energy computations and time delay during their processing in VANET. Therefore, we were motivated to join the vehicle to fog VM through a wireless vehicular communication network. Fog computing provides efficient task processing and scheduling capabilities between environments. Vehicular ad hoc networks (VANETs) are wireless communication networks facilitating data exchange between vehicles and infrastructure components. VANETs have gained significant attention recently due to their potential to enhance traffic safety and reduce traffic congestion. However, managing tasks in VANETs is challenging due to the networks’ dynamic nature and vehicles’ high-speed mobility. 

### 1.1. Fog Computing

**Figure 1:**
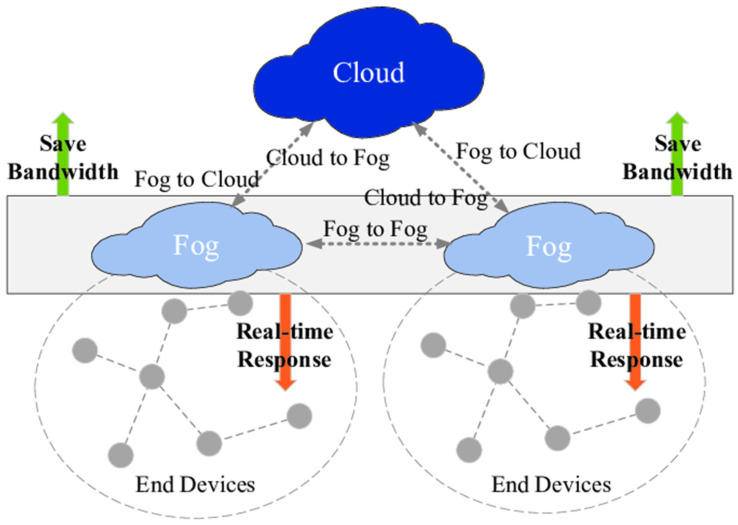
Fog and cloud computing platforms are connected with end devices for task management [4].

Due to fog availability, every node of the VANET can be linked and connected to the fog layer. Fog computing provides services like cloud computing near the users, which brings benefits like real-time data processing, real-time control, local data filtration and caching, visualization, lower latency, and data locality. In VANET, such collective processing and demand situations are now connected and submitted to the fog layer through fuzzy logic to save time and energy [1]. Fuzzy logic-based algorithms and emerging technologies provide optimal solutions for task management inside the loaded network. The fuzzy logic-based system provides a decision-based algorithmic technique that supports the extended and more effective way of task management in VANET. Typical fog architecture is displayed in Figure 1, in which every intelligent device of the IoT is linked with fog. The fog devices are connected to the cloud and another server in the environment [2].

Fog computing gains more popularity when cloud computing infrastructure is not feasible for providing services at the doorstep of VANET using IoT devices and sensors. In 2015, the number of sensors and IoT devices in VANET was 15.41 billion, which exceeded 30.73 billion in 2020 [3]. Figure 1 shows the cloud and fog computing platform connected to end devices. Fog computing enables us to provide the network connectivity of sensors and IoT devices at the network’s edge. It distributes at the network edge with the connectivity of heterogeneous devices in VANET. It also provides network and edge backup services. Edge computing provides the network structure needed to extend the capabilities of the cloud and make a more extensive resourceful network. Fog computing is a paradigm that enables computing resources and services to be located at the edge of the network, closer to the end user, rather than in centralized data centers. Fog computing is advantageous in VANETs, as it can help address latency, bandwidth, and network congestion issues.

Fog computing enables the cloud infrastructure to handle the task near VANET architecture to reduce processing time and energy constraints. Fog computing overcomes the security-related issues encountered in cloud computing [5]. The working of fog depends on intelligent gateways, which have more computation power and processing capabilities than the IoT devices. With fog and IoT devices, the bandwidth minimizes and enhances the fog devices’ performance. The servers in the fog are called cloudlets and are like central processing units (CPUs) with one or more than one graphic processing unit (GPU) working on the concept of batch processing. Fog computing uses minimal processing time and produces results with great efficiency because there is no burden on the central server. It also helps in geographically distributed locations where connections cannot be regular [6].

### 1.2. VANET and Its Architecture

**Figure 2:**
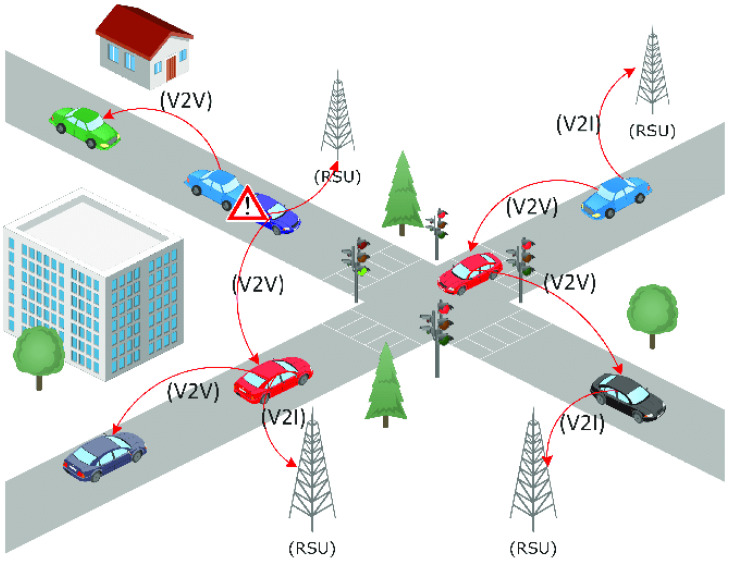
VANET environment and structure [12].

VANET is a network of connected computers that exchange data using vehicle to vehicle (V2V), vehicle to infrastructure (V2I), and infrastructure to infrastructure (I2I) communication models [7]. V2V communication forms a short-range wireless mesh network. Two or more vehicles directly communicate with each other to share messages and communicate data. In V2I communication, the vehicles forward or receive data from road infrastructures. V2I provides road safety and security. Meanwhile, in I2I communication, the RSU communicates with BS stations and other RSUs, and base stations communicate with road infrastructure. The most confidential and secure communication channel is used when communicating [8]. VANET is a remote arrangement implanted with the characteristics of an active topology, a massive and variable organized estimate, and portability. Figure 2 shows the structure of VANET with vehicle to vehicle (V2V), vehicle to infrastructure (V2I), and infrastructure to infrastructure (I2I) connectivity [9]. VANET is a subset of mobile ad hoc networks (MANETs) that shows vehicle communication using wireless technology [10]. These days, cutting-edge vehicles are prepared with present-day devices such as communication, tactile, and human interaction assets. The intelligent transportation system (ITS) uses VANET to enable the security policy and non-safety applications to decrease life risks and improve activity administration [11].

In comparison, the communication between vehicles utilized board units (OBUs) with physically collocated arithmetic units (AUs), based on IEEE 802.11p radio innovation. The OBU is a portable hub, and the roadside unit (RSU) is inactive in V2I communication. For trading data, RSUs can communicate with each other; this communication is called I2I communication. 

In VANET, task scheduling is one of the main issues due to mobility behavior, changing nodes, fast speed, limited capacity to process large tasks, and limited time constraints. The tasks are not adequately configured due to the RSU’s business. We encounter this when a task is loaded to the RSU, and then the RSU connects with the fog layer of the cloud. It takes time and energy to resolve the tasks of offloading and unloading. We skipped the RSU and directly connected the vehicle node with the fog to resolve this issue with a Wi-Fi connectivity sensor. The internet of vehicles (IoV) is the vehicular network with software, sensors, and technologies to exchange information [13]. The tasks are now loaded to the fog node with ever-changing concerns with the node processing capability.

### 1.3. Fuzzy Logic-Based Approach in VANET

**Figure 3:**
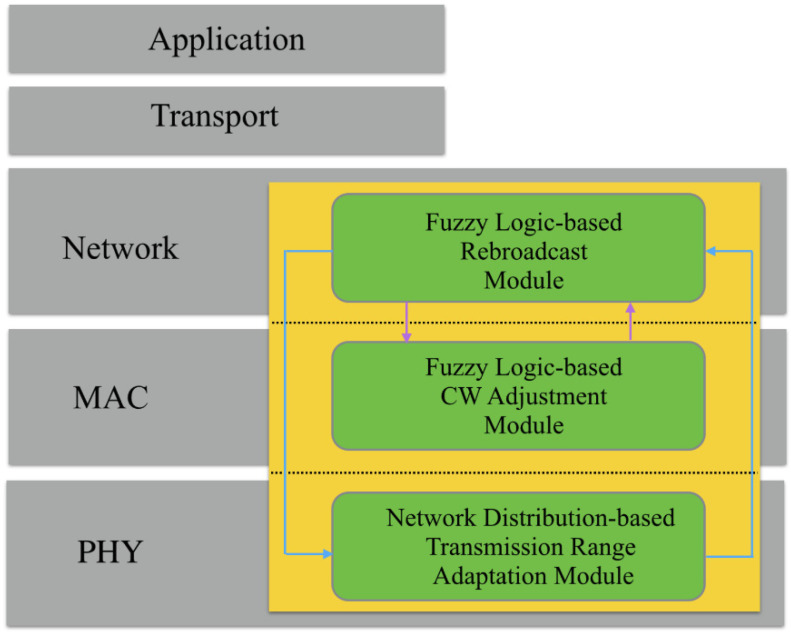
Working of fuzzy logic in VANET on network layers [15].

Fuzzy logic is a mathematical framework for reasoning with uncertain or imprecise information. Fuzzy logic has been used in many applications, including control systems, decision-making, and pattern recognition. The main reason behind using fuzzy logic is that it is compared with machine learning, and we applied a new approach to task scheduling. Much less work has been performed through fuzzy logic. Thus, we must prepare and test this. The result’s effectiveness is possible through our approach as we applied different techniques with different methodologies. Therefore, the results were improved compared with those in our selected base paper. The tasks are distributed to VMs in cloud computing to save memory and CPU processing in VANET vehicles [14]. A good cloud task distribution algorithm effectively maximizes the system resources and utilizes the task execution time for resource management. Task fairness is also improved due to the reasons behind the success ratio of the task distribution: the assignment of tasks to the fog virtual machine based on the fuzzy logic decision. The decision should be more effective and efficient under such a scenario. Figure 3 illustrates the use of fuzzy logic in the VANET environment. 

Task management in VANETs involves allocating resources and scheduling tasks for vehicles and infrastructure components. The task management approach proposed in this study is based on fuzzy logic and fog computing, and aims to improve the efficiency and reliability of task management in VANETs. The approach uses fuzzy logic to make decisions based on uncertain or imprecise information and fog computing to provide computing resources and services closer to the end user. In conclusion, the fuzzy logic-based task management approach using fog computing in VANETs is a promising approach that can help address some of the challenges associated with managing tasks in VANETs. By leveraging the benefits of fuzzy logic and fog computing, this approach can improve the efficiency and reliability of task management in VANETs, leading to safer and more efficient transportation systems.

On the other hand, the primary purposes of the task distribution at the edge are to reduce energy, ensure the task is completed on time, improve user experience, reduce energy consumption, and significantly improve power. Task scheduling is performed to complete work in a specified time with limited resources. With the increased amount of data to be processed, completing a task within a given time in fog computing is a significant challenge. Therefore, scheduling tasks and resources is a significant issue [16].

### 1.4. Motivation

In VANET, task handling and scheduling are required to schedule multiple processes for the task processors. Task scheduling in VANET is now one of the most effective ways to handle high computational and energy-consuming tasks. Task processing in the RSU involves calculating the time delay and energy consumed for task processing in VANET. Therefore, we were motivated to join the vehicle to a fog virtual machine (VM) through a wireless vehicular communication network. Fog computing provides efficient task processing and scheduling capabilities in the environment. Due to fog availability, every node of the VANET can be linked and connected to the fog layer. The fog is one of the parts of cloud computing that process tasks in VANET effectively.

In VANET, collective processing and demand situations are connected and submitted to the fog layer through fuzzy logic to save time and energy [17]. Fuzzy logic is considered one of the techniques for artificial intelligence, where the intelligence is achieved using fuzzy classes under some parameters (in this case, utilization of CPU, storage, bandwidth, task deadline, latency, and task execution time). The fuzzy logic rule is understandable and easy to deploy under these conditions. The fuzzy logic-based technique suits this scenario because there is no separation of the classes based on the features that are vaguely defined. The fuzzy logic approach is most suitable. Therefore, based on this discussion, fuzzy logic is suitable for our approach to task management under a fog environment for VANET. In VANET, efficient task handling and scheduling for energy-consuming processes are crucial. The RSU’s task processing in VANET prompted our interest, leading us to explore vehicle to fog VM connectivity via a wireless vehicular communication network. Fog computing, an integral part of cloud computing, optimizes task processing and scheduling in VANET, leveraging fuzzy logic for collective processing. Fuzzy logic, an AI technique, suits the vaguely defined VANET features, making it apt for task management in a fog environment. Fog computing enhances VANET task processing by bringing resources closer, reducing latency congestion, and improving response times for real-time applications. Utilizing fog nodes at the network edge enhances the overall performance of VANETs.

Fog computing enhances the task processing capabilities of VANET (vehicular ad hoc network) vehicular tasks by bringing computational and storage resources closer to the network edge, reducing the latency and congestion associated with sending data to the cloud for processing. This results in improved response times for real-time applications and enables the processing of large amounts of data generated by vehicles in a VANET. By utilizing fog nodes located at the network edge, vehicular tasks can be processed faster and more efficiently, which can improve the overall performance of VANETs.

### 1.5. Importance of This Research

The data produced by urban vehicles continue to accumulate in nature, which allows us to predict road safety and traffic control. Indicating the speed of metropolitan areas’ vehicles is challenging [12]. VANET connectivity with fog nodes is one of the most enhanced and efficacious methodologies to provide the demanded and efficient processing through proper implementation. In VANET, task scheduling is one of the main issues arising from mobility behavior, changing nodes, fast speed, limited capacity to process large tasks, and limited time constraints. In addition, one of the reasons for this is that connecting a job from the RSU to the fog layer of the cloud takes longer and requires more energy to complete the offloading and unloading process. As a result, we removed the need for RSU processing and instead connected the vehicle directly to the fog layer. The tasks are now loaded directly onto the fog node, with consideration for the node’s processing capability, which is constantly changing. We employed a fuzzy logic-based task scheduling technique to schedule the jobs to the fog. Fuzzy logic is a more straightforward and efficient approach to decision-making for tasks on VANET nodes. 

### 1.6. Problem Description

In VANET, task scheduling is one of the main issues due to mobility behavior, changing nodes, fast speed, limited capacity to process large tasks, and limited time constraints. The tasks are not adequately configured due to the RSU’s business. The RSU is not a cloud-based platform and has limited task scheduling and processing resources. Moreover, time and energy issues arise in RSU-based task scheduling. The limited capabilities of the RSU cannot manage the task processing capabilities of vehicles in VANET.

In this paper, we considered the below-mentioned questions during the task scheduling.

(i) How does fog enhance the task processing capabilities of VANET vehicular tasks?
(ii) How does one schedule the tasks through fog computing to save time and energy consumption from one node to another?
(iii) How does fuzzy logic enhance task scheduling using fog computing?

### 1.7. Research Contributions

The proposed system’s main contribution is task management and data handling with VANET. Specific contributions are listed below.

- In our proposed technique, we proposed a fuzzy logic-based fog-enabled task scheduling technique to enhance the task time and energy consumption efficiency in VANET.
- This research provides that fog enables fuzzy logic-based task scheduling that minimizes the delay rate in task processing and migration to fog VMs.
- The proposed technique uses vehicle nodes connected with VANET architecture consisting of fog and fuzzy logic fuzzification methods to offload the resource-intensive tasks to the fog layer through the scheduler and classifier.
- The proposed model was evaluated using the Mumdani fuzzification method to utilize the fuzzy logic under the supervision of the classifier to overcome the response time and service delivery time in the VANET environment.
- We designed a V2V-based fuzzy logic-based VANET architecture in which the vehicles are directly connected with a fuzzy classifier to overcome the energy consumption and response time for tasks produced in VANET.
- The proposed algorithms selected the tasks from the environment based on their features and matched them with the threshold. They passed them toward the fuzzy logic engine to be decided under the supervision of the fuzzy logic-based system.
- The proposed technique effectively schedules all the tasks through fog-enabled VANET.
- By contrasting it with current methods, the simulation experiments demonstrate the effectiveness of the suggested task scheduling framework. According to the findings, the suggested system performs admirably in terms of task ratio, typical turnaround time, makespan time, and average latency within the constraints of the network.

The rest of this paper is organized as follows. Section 2 presents the related work on fuzzy logic-based models to improve task management in VANET. In this work, we study the state-of-the-art literature review, define the study resources that we enlist, provide justified background knowledge related to the current context, and enhance the proposed scenario with the proposed methodology. With edge computing integration, fuzzy logic-based systems are discussed to support task processing on the edge cloud. The state-of-the-art literature is discussed and it is proposed to view the resultant values from the literature and enhance the working experience. Based on the current parameters, the literature articles do not present the time and energy efficiency our approach achieved. Then, in Section 3, we outline our approach for the research and propose solutions to the relevant problems encountered in Section 1 of the paper. The proposed system effectively distributes the tasks toward the fog layer using the fuzzy logic system by applying the Mamdani fuzzy logic system. The tasks are distributed based on the utilization of CPU, storage, and bandwidth requirements. The latency, task execution time, and task deadline time are considered for the final considerations. Then, Section 4 presents the proposed method with simulations using a fuzzy logic-based approach. The results effectively show the improvement in task scheduling from previous state-of-the-art techniques. Section 5 concludes the description of our proposed system, highlighting that our technique is straightforward for fault tolerance methodology. 

## 2. Literature Review

In the literature, several decision algorithms have been proposed. Multiple decisions and related parameters are assigned and provide the extension inside the fog and other associated parameters like task deadline and execution, security, the CPU time required, and processing. The limited resources available at the fog node do not provide further enhancements at the fog with the ability to control and coordinate the available resources. Moreover, the resource requirement demands the fog cannot correspond to and handle the task requirements of the vehicles at VANET. Infrastructure-assisted job scheduling is proposed with RSU-based volunteer computing. It utilizes surplus resources and enhances the enhancement mechanism [18]. Many fuzzy logic systems use work-based task processing in fog environments. This paper focuses on fuzzy logic-based task scheduling and processing in terms of fuzzy logic-based task optimization and processing capabilities. Due to the processing and computational power, the fuzzy system is always applicable for decision-making and efficiently handling network requirements. The parking edge computing idea is proposed via adopting the edge server’s task handling mechanism [19]. Fog with fuzzy logic effectively handles the changing demands of tasks’ dynamic nature for task scheduling. This motivation helps us to propose a decision algorithm using fog and fuzzy logic-based infrastructure to schedule the tasks toward the fog environment with network latency, resource requirements, and time constraints. The task requirements in VANET continuously extend the task processing capabilities at different nodes with the ability to control and coordinate the processing time. The task assigned to processing tasks toward the fog nodes has the ability to coordinate and extend the processing capabilities of these nodes with the fog layer.

It is the methodology to offload the tasks toward the fog layer of cloud computing. The transmission offloading is proposed as a data transmission scheduling consideration broken point continuingly transferring technique (DTS-BPCT) to distribute the contents in the VANET. The relay nodes are considered to transfer the data. The computational layer at the fog provides the processing and storage capabilities to send the tasks back to the vehicle. Task scheduling offers limited use of battery lifetime, power scheduling, energy-aware communication, bandwidth handling, and time consumption. The task offload loading reduces the requirements of the vehicles. Task scheduling overcomes the communication cost and energy challenges. The scheduling is handled through an integrated programming model that provides reliable and efficient data efficiency and transparency [20]. The task scheduler is part of the task scheduling that offloads the tasks toward the fog layer and then keeps track of offloaded tasks. The decision is based on task execution time, network bandwidth, memory, upload time, processing energy, local execution cycles, and allocations of resource parameters. Task scheduling is one of the leading research objectives in this research.

Task scheduling studies have been investigated for processing capabilities in collective fog and cloud environments. The authors in [21] propose a new heuristic-based task scheduling algorithm in fog computing to balance the cost and makespan time parameters. The MANET standards were adopted to access the routing-based task scheduling policies. Deadline-based scheduling and hybrid scheduling were adopted. Moreover, the authors in [22] are focused on the communication cost and makespan time for handling the tasks with the fog resources. An adaptive double fitness genetic algorithm was proposed, named ADGTS, for task scheduling and compared with the Min–Min traditional algorithm. The algorithm defines the relay’s boundary to minimize the requirements for multi-hope placement techniques. 

The authors in [23] executed the local device tasks and offloaded the task toward the edge or fog when the local device failed to execute. The heterogeneous VMs are essential inside the fog to schedule the tasks and improve overall system efficiency. To handle such a scenario, the authors used Fog Brothers to control the fog layer, using fog resources to schedule the tasks. The scheduling depends on the delay tolerance and availability of the tasks to be offloaded. 

Another contribution by the authors in [24] involves using fog to enable cloud task scheduling techniques. The technique schedules the manufacturing tasks toward the fog or cloud resources. Virtualization technology is adopted to handle these tasks for task processing capabilities. The proposed task scheduling mechanism is adopted to schedule and process these tasks toward the fog cloud containers. The contributions are to reduce the makespan time after improvements in the task processing capabilities of the fog nodes. The cost of communication, makespan, and processing power of the fog–cloud environment is considered using energy-efficient task scheduling techniques. 

The authors of [25,26] proposed scheduling and prioritization algorithms in VANET. The algorithms are smallest data size first (SDF), first in, first out (FIFO), maximum quality increment first (MQIF), longest wait time (LWT), least selected first (LSF), maximum request first (MRF), longest stretch first (LSF), and first deadline first (FDF). The LWF gives higher priority to task processing and task enhancement capabilities. The highest level of priorities is assigned with various messages. In FDF, the scheduling is performed using messages using their deadline techniques. In this technique, the fuzzy logic systems are implemented to propose a system development time, energy, and resource utilization, but the results show limitations in the constraints’ dependence. In [27], the SDF is shown to handle the highest-priority tasks first and lower-priority tasks second for scheduling. The highest-priority tasks are considered using the fuzzy logic computational system to get offloaded. Still, this approach’s limitation is that lower-priority tasks require more prolonged periods. 

The authors in [28] proposed a job-shop scheduling technique using a multiagent-based system for intelligent environments in VANET. Inside the industrial VANET, the fog nodes handle their task requirements. The energy consumption problem is considered in this research. This research proposes the energy-aware load balancing and scheduling (ELBS) technique. Initially, the authors built a fog-based model for handling power requirements between the workload and task processing. Secondly, load balancing and optimization were performed to handle these tasks for processing. The particle swarm optimization algorithm was adopted to use the optimal solution. 

Another study [29] focused on task scheduling in heterogeneous computing environments to handle the proposed systems’ energy requirements. The proposed data efficient beware task scheduling (DEBTS) algorithm was implemented to handle the delay energy constraints using the Lyapunov optimization algorithm (LOA). The algorithm results were compared with traditional random scheduling algorithms and showed effective results compared with the existing techniques. Another fuzzy logic-based task scheduling technique is proposed in [30]. The authors use practical task scheduling algorithms using fog in VANET. The constraints are used to help with the energy efficiency and task processing capabilities for the task processing. The proposed technique handles the task and cost constraints in VANET task scheduling. The resources are compared with the neighboring environment. The ability to handle the practical energy constraints results in the provision of the signal settings and enhances the selection criteria. The total energy is utilized to handle the task processing and provides the complete criteria for the task scheduling techniques.

Moreover, the authors in [31] propose a first-time operation (FTO) algorithm for fair task scheduling. It selects the fog nodes for every vehicular request and provides and maintains the delay-sensitive task processing and corresponding energy requirements for task scheduling. The nodes selected based on the scheduling provide the knowledge to discover the wide range of applications for scheduling on the cloud using the FTO algorithm. The algorithm works fine, but the dataset adopted does not take into consideration the energy constraints. 

However, the VANET environment always requires special attention for task processing due to the limited capabilities of its tasks, which are processed on local or remote machines. Congestion is established whenever the ratio of offloaded tasks is high. The problem of task scheduling and processing always requires particular attention for task processing [32]. The control parameters that result in less delay-sensitive task processing are performed using these limited-capabilities requirements. The strategies are implemented to offload the tasks using fog-enabled RSU-based techniques. Although effective results are produced, the limitation of the method is the overhead time when the RSU is involved, which delivers extra time in terms of the task processing capabilities. Therefore, to handle these operations, we must maintain the task scheduling and processing capabilities for the task handling. 

The authors in [25] proposed a fog virtual machine-based hybrid optimization scheduling algorithm. The authors use fog-based VMs to schedule the tasks toward the fog cloud. Network Simulator 2 (NS-2) simulates energy efficiency, routing, and load balancing results. Whenever the tasks in the proposed approach increase, the performance of the proposed method is also increased. The simulation parameters achieve the highest results based on user predictions and enhance the working experience. Fog in VANET is one of the most demanding ways to fulfil the vehicle’s task processing demands. Vehicular fog and traditional vehicular communication paradigms extend the fog standards paradigm. Several IoT devices are implemented using vehicular communication architecture. The virtual machine plays a significant role in this contribution. The authors of [33] present fog computing-based VANET architecture inside an informative application scenario. The article presents the multiple benefits of cloud-based platforms. The vehicles used in task processing, task management, and cloud upload make the system more efficient. Still, their reliable and effective features enhance their core contributions. The storage and onboard computational resources and communication resources are underutilized.

The state-of-the-art literature from the most recent and advanced resources was researched. Based on the literature, it was concluded that current research is performed for handling task scheduling and management using fog, edge, and cloud computing. CPU utilization, energy efficiency, memory/storage, bandwidth utilization, and task management are the key parameters that the experts consider in their current research. The limitations defined in this research enable us to provide a complete and robust fuzzy logic-based task management mechanism using fog resources and cloud edge-enhanced parameters. After considering the limitations of the current techniques presented in the literature, we define the state-of-the-art techniques to be presented in this context for the final utilization of the resources. 

In vehicle ad hoc networks (VANETs), several studies have proposed using fuzzy logic for task scheduling. These studies aim to improve the efficiency and performance of VANETs by using fuzzy logic to make decisions about allocating resources such as bandwidth and processing power. The fuzzy logic-based task scheduling algorithms consider various factors such as traffic congestion, network load, and priority of tasks to make decisions about scheduling tasks in the network. The results of these studies have shown that fuzzy logic can effectively improve the performance and efficiency of VANETs. However, the precise details of these studies can vary. The specific design and implementation of a fuzzy logic-based task scheduling algorithm depend on the specific requirements and constraints of the VANET in question. The following points show the conclusions drawn from this study.

- Multiple decision algorithms have been proposed in the literature for fog-based task scheduling, considering parameters like task deadline, execution, security, CPU time, and processing.
- Infrastructure-assisted job scheduling using RSU-based volunteer computing has been suggested to utilize surplus resources and enhance the overall system performance.
- The transmission offloading technique (DTS-BPCT) is proposed to distribute contents in VANET, considering relay nodes for data transfer and computational layers at the fog for processing.
- Various heuristic-based task scheduling algorithms, including deadline-based and hybrid scheduling, have been proposed to balance cost and makespan time parameters in fog computing.
- Fuzzy logic-based task scheduling algorithms have been explored to handle the dynamic nature of tasks in VANETs, considering factors like delay tolerance and availability of tasks.
- The literature emphasizes the significance of fog-enabled task scheduling in handling energy constraints, communication costs, and processing power for efficient task processing in VANETs.

In summary, multiple techniques proposed in the literature are used to deal with and provide the solution for VANET task scheduling. In the deeper analysis, we observed that many techniques adopted to provide the solution are based on the tasks under cloud, fog, and edge environments. Although their statistical and computational results are better, their fuzzy logic-based techniques lack time and energy under VMs and task processing ratios. This is one of the research gaps still faced during the processing period in terms of the ability to concentrate and enhance the processing capabilities. However, some techniques adopt fuzzy logic under the edge or in cloud computing. Still, they fail to address the core results, i.e., the makespan time, average processing time, task completion time under both VMs’ use, and number of tasks. Some techniques involved adopting the fog-enhanced layer to schedule the tasks, but this brings the issue of extra computations served during task offloading, which burdens task migration and computation. SDF, FIFO, MQIF, LWT, LSF, MRF, and FDF are the techniques that worked with the same concept. Still, they face the issue of extra computations during migration and power consumption in these computations, leading us to design a new proposed FETS model to address these limitations and provide a reliable time management and energy-efficient solution under this environment. The proposed technique is described and explained with algorithms in the next section to explain the required concepts and effective mechanisms. Table 1 provides the literature review in summary form and a reliable literature review structure.

## 3. Methodology

**Figure 4:**
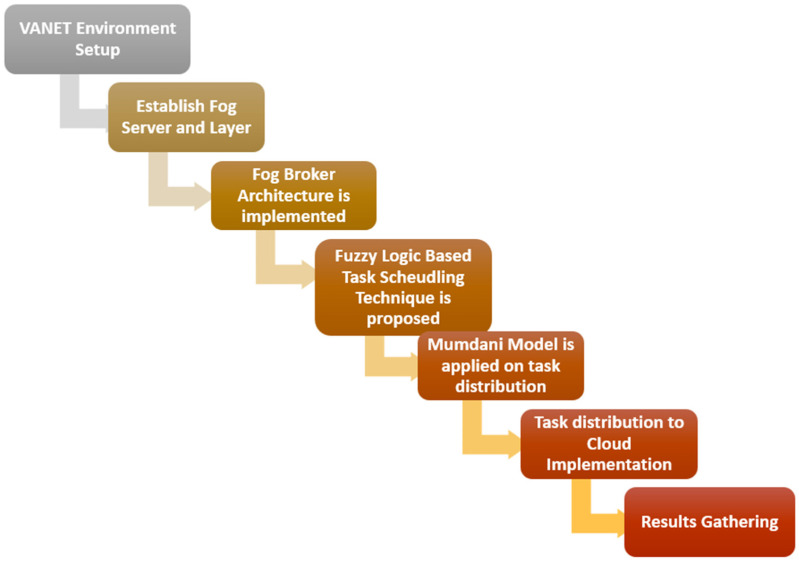
Workflow diagram of proposed methodology.

We present a fog-based proposed framework for task scheduling in mobile vehicular ad hoc networks (VANETs). In this section, we also explore the problem statement with the solution through a fog-enabled task scheduling framework to schedule the tasks using a fuzzy logic system. The critical points of the proposed techniques are illustrated in the following points. Also, Figure 4 shows the workflow steps adopted in the proposed methodology.

- Initially, we set up the VANET environment and connected with fog to enable cloud architecture.
- Fog broker architecture was proposed to help collect resource allocation and task scheduling information.
- Fog server VMs were set up to execute the scheduled tasks in the fog environment.
- A fuzzy logic-based task scheduling technique was proposed to adopt accurate and effective resource utilization features.
- The scheduler was proposed and connected with a classifier and fuzzy logic system for efficient and effective resource distribution mechanisms.
- The Mamdani model was applied to fuzzy logic-based task distribution decisions. A decision algorithm was applied for fuzzy logic-based task distribution.
- The final decision for task distribution toward the cloud was made using the fuzzy logic-based system and its implementation.

### 3.1. Fog Cloud Architecture

**Figure 5:**
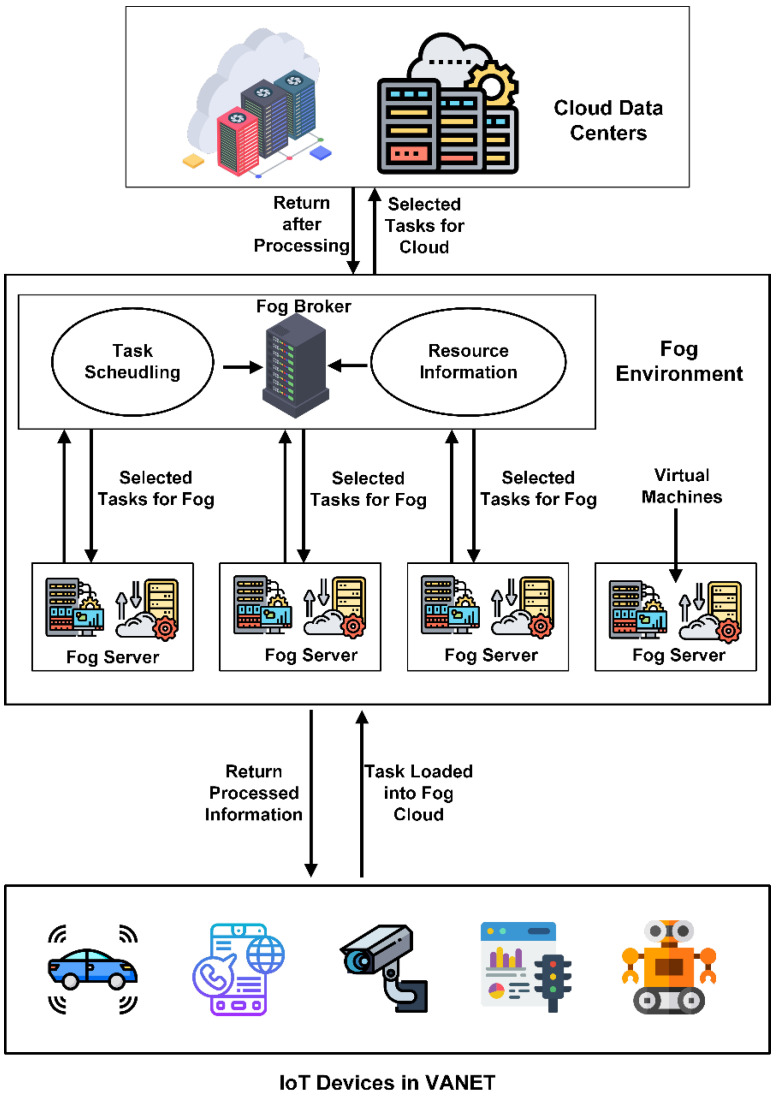
Fog computer architecture with task scheduling framework.

Fog enables the applications to execute near the vehicles. Figure 5 shows the fog-enabled cloud-based architecture framework for enhancing task decision-making. The end user submits the task from vehicles and IoT devices. In this research, we used the vehicle scenario in the VANET environment, where the vehicles transmit their tasks toward the fog layer. The fog layer of our proposed approach consisted of one set of fog servers with limited access to the network resources. The proposed fog servers use their VMs for processing and task management. The network resources in the VANET are not used by the fog itself, which is a very effective way to utilize the network with limited access to VANET network resources from the fog layers. Still, these resources are enough for the task processing of vehicles. The fog also contains virtual machines (VMs) to provide connectivity with the fog layer. The fog server receives the tasks from the vehicles and forwards them to the fog broker. The broker is responsible for managing the services originating from the fog resources. The fog scheduler is accountable for scheduling the fuzzy system’s tasks to the fog layers for further processing at their layers. After receiving the tasks from the fuzzy log defuzzification system, the fog scheduler selects the VM. The scheduling queues are maintained on the fog layer for efficient resource provision and handling. On the other hand, the cloud layer consists of powerful computers called data centers. These cloud centers process tasks of very high latency, like video processing and other highly data-intensive tasks beyond the fog servers’ limit. 

In this work, we considered that the tasks received at the fog broker are independent, non-preemptive, and inseparable. Let us consider that T is the entire set of tasks received at the resource broker of the fog layer. Every task has a unique identification number, number of instructions inside the task, size of the task before and after results, and task deadline. 

The CPU rate and task execution level during the workflow of the task processing are presented. The VMs are considered heterogeneous, apart from their physical and logical structure. V is the total set of VMs that exist on the fog server. Therefore, every VM has a unique identifier, standard type, bandwidth, storage, and existence. The fog server hosts multiple VMs based on the fog servers’ processing power and resource-handling capacity.

This paper focuses on minimizing the processing time of VANET tasks using fog and fuzzy logic for task scheduling in VANET. The processing time is considered using the following time activities.

- Task transmission delay time.
- Task submission from vehicle to fog VMs.
- Fog VM execution time for individual tasks.
- VM capacity for timely processing.
- Time for result generation and reception at the vehicle.
- Time for fog reception toward the fog layer-based task processing at the cloud layer.
- Makespan’s time from task submission to results it is capturing.

After receiving the tasks, the fog broker processes the task on a fog VM or cloud server. The decision is made based on the task’s characteristics. A function is executed for task processing for the selected fog VM or cloud server. This is the first function after the performance. The second function is the optimization scheduling for selecting the fog layer and VM for the scheduling process. The turnaround of the task was considered and assigned to the fog VM.

### 3.2. Proposed Fuzzy Logic System for Task Scheduling

**Figure 6:**
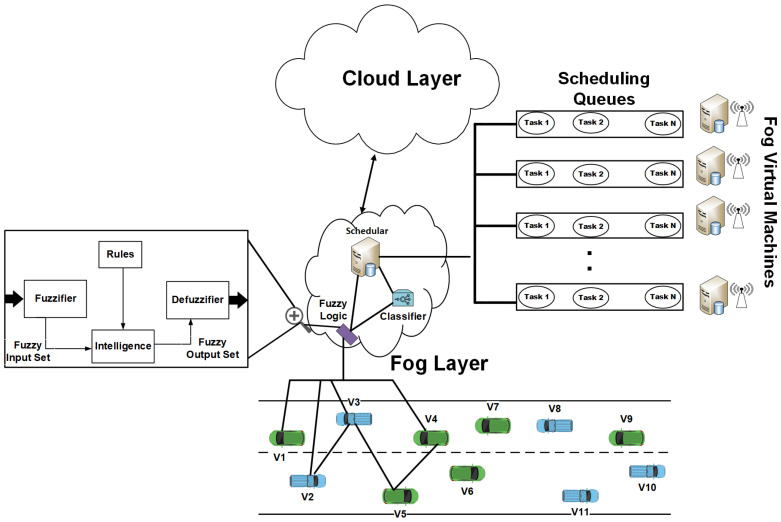
Proposed fuzzy logic system for task scheduling in an IoV-based VANET approach.

In this section of the paper, we determine that the fog enables task scheduling using the proposed environment’s fuzzy logic system. This is the paper’s first step, which is executed for task scheduling. The fuzzy logic system selected and decided on the task scheduling on the fog VM or cloud server. In the second step, we present the real-time fuzzy logic-based task scheduling system to choose the best available VM from the vehicles for task scheduling and processing. Figure 6 shows the proposed system with fuzzy logic, cloud, and fog servers in the VANET. The process of performing the fuzzy logic task schedule is explained in the steps below.

- Initially, the vehicles move in one direction down the highway and connect using RSU-based clusters.
- Vehicles possess limited memory, processing power, storage, bandwidth, and resource allocation, so the heavily loaded tasks require processing through the fog layer.
- Before the tasks are sent to the fog layer, the fuzzy logic system and scheduler classify tasks that require uploading toward the fog and cloud.
- Algorithm 1 decides on the scheduling decision from the scheduler, and Algorithm 2 decides on the fuzzy logic system using the Mamdani model.
- After selecting the tasks for fog, the fog broker checks for the available VMs on every fog layer and assigns the tasks based on matching bandwidth, processing power, memory, and storage requirements.
- The fog VM executes the tasks and returns the solved information to the fog broker/scheduler.
- The fog broker/scheduler assigns the information to the vehicle concerned for further action.
- If the task is too heavy and does not meet the requirements of fog VM, the scheduler schedules the tasks to the cloud layer for processing.

#### 3.2.1. Decision Algorithm Using Fuzzy Logic

The algorithm provides the sequence of steps for fuzzy logic-based task scheduling decisions. The scheduling decision algorithm considers the scheduling tasks using resources and process demands. Time constraints are also considered for scheduling. Resources are available on the fog VM, server, and latency between fog and cloud. We compute the minimum values for the resource utilization from the resources and provide these to the cloud environment for efficient task processing. The CPU rates are also the minimum and maximum resources for scheduling tasks. The bandwidth and storage capacity of the fog servers are also considered in the fog environment with effective and efficient resource utilization privileges. Although the resources are appropriately utilized after the task is submitted to the fuzzy logic system, the tasks are distributed with values such as stage, CPU, and bandwidth consumed. Based on this information related to the tasks, the constraints that exist alongside the tasks are network latency and the deadline for fog and cloud environments. We adopted the min–max normalization method to find the minimum values for the fuzzy logic system. We utilized the trapezoidal and triangular member functions for the practical membership values for the final output of the functional requirements. The proposed system presents a novel approach for task management in vehicular ad hoc networks (VANETs) by utilizing fuzzy logic and fog computing. By incorporating fuzzy logic, the system can handle the uncertainties and imprecisions associated with VANETs, making it more robust and reliable. Fog computing allows real-time decision-making and task allocation, which is crucial for VANETs, where time-critical tasks must be executed promptly. The proposed system was evaluated using simulations and compared with existing approaches, showing improved task completion rate and response time performance. Overall, the proposed approach has the potential to enhance the efficiency and effectiveness of task management in VANETs, thus contributing to the development of intelligent transportation systems.

The dataset shown in Table 2 contains parameters using crisp values. These values are converted into linguistic membership functions for system output. System utilization, latency, and task deadline are the main crisp parameters and are transformed into the final decision, i.e., task execution, using the longitudinal membership function. Task execution is the final output from a system that decides to offload tasks. In Table 1, we show the crisp input and crisp output parameter values. Table 3 defines the fuzzy set rules for the system’s functionality for input/output variable values. 

**Figure 7:**
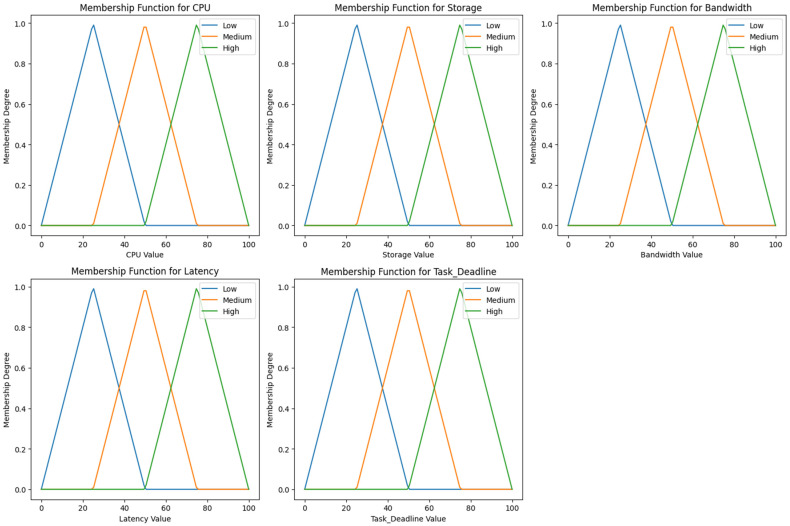
Fuzzy set input and output variables with membership functions.

**Figure 8:**
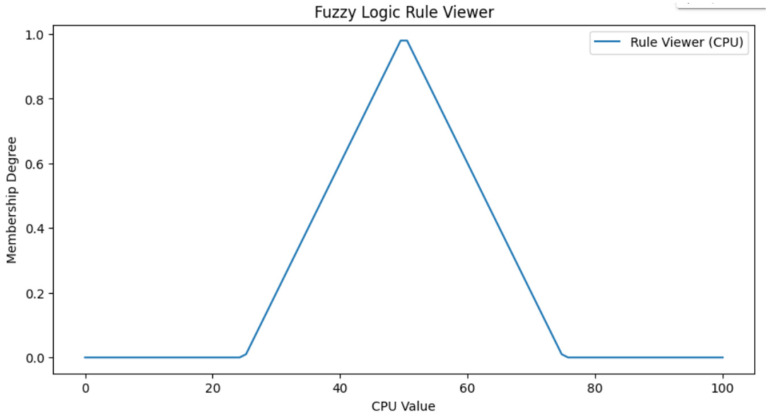
Rule plot for the CPU value with membership function.

**Figure 9:**
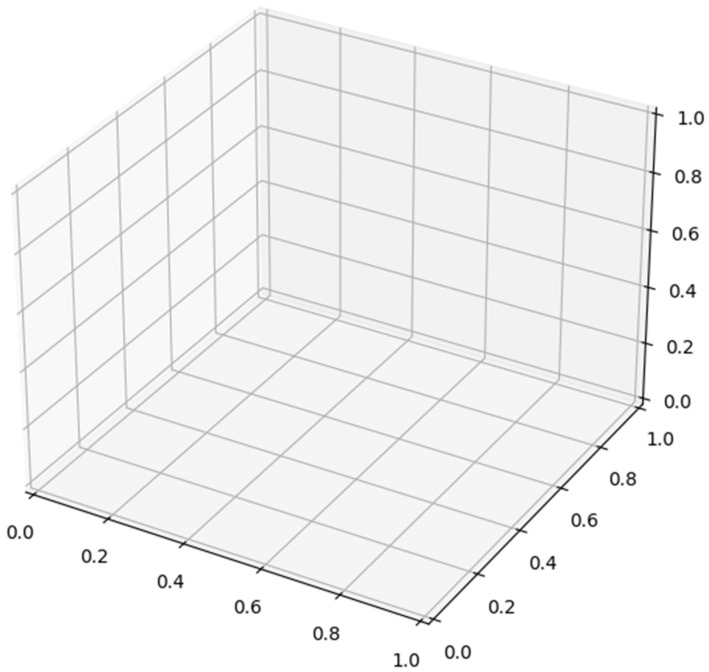
Surface viewer plot for fuzzy set of values.

Based on the values assigned to the fuzzy logic, the crisp values with fuzzy set membership functions are defined in Figure 7. We generated 486 logic rules for the fuzzy logic system and one output variable based on the five inputs in the fuzzy input variable. Table 2 shows the rule-based sample based on the five input values. In this system, we adopted the Mamdani model with the system. The adaptation of the Mamdani fuzzy inference system is that it obtains the rules, and according to these rules, the values are associated with the rules. Once the fuzzification is performed on the input set from Table 2 and Table 3, we adopt the center of gravity defuzzification method to find the output set of values. Table 3 shows the notations used inside the manuscript with some description values for the proposed Algorithms 1 and 2. Algorithm 1 is a decision algorithm that offloads the task to the cloud or fog nodes. Algorithm 2 performed the fuzzification and defuzzification process. Figure 8 and Figure 9 show the rule and surface viewer plots for the fuzzy set membership functions.
**Algorithm 1:** Decision algorithm based on scheduler and fog broker**Input:** CPU utilization, bandwidth, storage, task deadline, network latency, fog VMs, cloud resources **Output**: Task scheduling initial decision Steps: 1.    $Node\;Mi\;receive\;a\;new\;packet\left(packet_{id},\;packet\right)$ 2.    Get GPS coordinates 3.    If (packet direction is from the back) 4.       $check\;hop\;count\;i.e.,\;\left(n=ni\right)$
5.         If (n <= 5) 6.             waiting_time() ← compute() 7.                 If (initially receipt of packet i.e., packet_id = $id_{i}$) 8.                   set (waiting time) ← lower_limit() 9.                 else 10.                   increase(packet reservation ratio) ← packet_id 11.         expire(waiting time, vehicle $m_{i}$) 12.             If (waiting time(packet_id) ← min_reserved() 13.                   Get_info(neighbour node, GPS) 14.                 Packet_forward(location(previous forward, packet_id = $id_{i}$) 15.                    If (n <= 4) 16.                       forward(vehicle-front, same direction) 17.                    else (n == 5) 18.                         forward(alternate-path) 19.                 else (packet_id = $id_{i}$ ≠ min-waiting-time) 20.                    drop(packet_id = $id_{i}$) 21.             else(n > 5) 22.               drop(packet_id = $id_{i}$) 23.          else 24.              drop(packet_id = $id_{i}$) 25. End

In the algorithm, the decision threshold values are given through decision parameters. The storage, deadline, bandwidth, and CPU of every task from vehicles are the input values, and the decision of the task is the output from the function. Adopted trapezoidal and triangular membership functions are used to determine the membership values. The algorithm chooses the scheduled task based on the defined threshold values. The task offloading is provided based on the layer-based architecture scheme. The objective of Algorithm 1 is scheduling decision-making using a fuzzy logic system. Algorithm 1 is required to achieve the selected decision-making technique. 

#### 3.2.2. Fuzzy Logic-Based Decision Algorithm

Algorithm 2 is applied when tasks arrive on the fog scheduler/broker layer queue. A fuzzy logic-based decision algorithm executes real-time task scheduling. If the required resources are not provided, all the tasks are shifted toward the cloud, and if the resources are provided, then the tasks are scheduled using the min–max normalization technique. Algorithm 1 decides the task for forwarding to the cloud or fog VM. The tasks are ordered in a sequential manner using a queue called the fog queue. All the tasks are initially ordered in ascending order using queue operations based on their deadline time. Algorithm 2 shows the complete functionality of the proposed system.
**Algorithm 2:** Fog/cloud assignment of tasks using fuzzy logic-based decision algorithm**Input:** T, V, NL, Q, FN (From Table 3) **Output:**$\;Z_{T}$ (fuzzy logic output using Mumdani model for fog/cloud task assignment) Steps: 1. $for\;\left(T\;to\;Q\right)\;do$ 2. $if\left(resources\;\neq fog\right)\;then$ 3.    $T_{forward}\;\to \;\;fog\_Cloud()$ 4.  else 5.        $determin\left(c,\;s,\;b\right)$ 6.        $normalize\left(c,\;s,\;b\right)$ 7.        $decision\left(c,\;s,\;b\right)\;\to \;FLDA_{c,\;\;s,b}$ 8. Algorithm 1 9.          $if\left(decision\;\to fog-cloud\right)\;then$ 10.             $forward_{task}\left(cloud\right)$ 11.     else 12.           $insert_{task\;}\;\to FQ$ 13.           $order_{tasks}\left(asc\right)$ 14.     $for\left(task\;t_{i}\;in\;FQ\right)\;do$ 15.      Initialize VM ($VM_{0}\ldots ..\;VM_{n})$ 16.              Calculate resource utilization. 17.            Obtain the least-load VM. 18.     End for 19.      $forward_{task}\;\to \;\;VM_{resource}$ 20.  End For 21.  End If 22.  $get_{cloud}\;\to \;\;results\left(fog/cloud\right)$ 23.    $compute\left(\right)\;\to \;\;T_{proc\;}\;\left(Total\;Processing\;Time\right)$ 24.    $if\left(T_{proc}\leq \;dl\left(T\right)\right)\;then$ 25.      $z_{T}\;\to 1$ 26.    else 27.      $z_{T}\;\to 0$ 28.    End if 29. Return ($z_{T})$


Algorithm 2 defines the final decision after passing from the fuzzy logic scheduler. After the algorithm’s execution, the tasks are distributed toward the fog layer after passing from the scheduler. The VM utilization is observed using the cloud-based scheduling technique to enhance the proposed algorithm’s decision-making process. 

Mamdani fuzzy inference is a method of reasoning based on fuzzy logic that uses if–then rules. The Mamdani fuzzy inference system includes fuzzification, which shows input variables are fuzzified to determine their degree of membership in fuzzy sets. The degree of membership is typically represented by membership functions, often triangular or trapezoidal. Mathematically, for a single input variable *X*, we have Equation (1).

(1) $$Membership\left(X,\;A\right)={µ}_{A}\left(X\right)$$

where *A* is a fuzzy set, and ${µ}_{A}\left(X\right)$ is the membership function of *X* in set *A*. Then we have rule evaluation, which evaluates the antecedent (if-part) of each rule by combining the fuzzy sets using logical operators (usually “and”). The degree of fulfilment of each rule is determined. Equation (2) shows rule *R*.

(2) $$Rule\_Strength\left(R\right)=\min({µ}_{A_{1}}\left(X_{1})\right),\;\min\left({µ}_{A_{2}}\left(X_{2}\right)\right),\;\ldots .,\;\min\left({µ}_{A_{n}}\left(X_{n}\right)\right)$$

where $A_{i}$ is the fuzzy set in the antecedent of the rule and $X_{i}$ is the corresponding input variable. Then, we have an aggregation function that combines the rule strengths to obtain an aggregated fuzzy output set using Equation (3).

(3) $$Aggregated\_Output\left(Y\right)=max\;(Rule\_Strength\left(R_{1}\right),\;(Rule\_Strength\left(R_{2}\right),\;\ldots .,\;(Rule\_Strength\left(R_{m}\right),$$

where *m* is the total number of rules, and the last step is defuzzification, which transforms the aggregated fuzzy output into a crisp output using Equation (4).

(4) $$Crisp\_Output\left(Y\right)=\frac{\int Y.Aggregated\_Output\left(Y\right)dY}{\int Aggregated.Output\left(Y\right)\;dY}$$

The equation represents the centroid method, where *Y* is the output variable.

#### 3.2.3. Complexities of Algorithms

Detailed in the study findings, Algorithm 1 is a machine learning decision-making algorithm that includes training and testing resources. The technique focuses on microservice-based work scheduling toward the mobile cloud by developing and testing machine learning models. The decision-making model training in this process is guided by qualities found in the data, which act as the source. Algorithm 1’s complexity is O(log n), which denotes a logarithmic growth pattern concerning the volume of the input data. After being educated by the machine learning algorithm, Algorithm 2 accepts the tasks and directs them into the decision-making server for the ultimate migration to mobile cloud-based virtual machines (VMs). Algorithm 2’s complexity is O(n log n), which denotes a linearithmic development pattern depending on the processing power needed for scheduling tasks. These complications convey the algorithms’ scalability and efficiency concerning the required input and computational activities.

## 4. Results and Discussion

This paper describes the proposed system’s performance evaluation through a simulation environment. The proposed method is simulated through the provided iFogSim simulation technology.

### 4.1. Scenario

We implemented the proposed methodology in the iFogSim simulator to implement and evaluate the proposed method and algorithm. iFogSim is built on CloudSim simulation technology and coded in the Java programming language. We adopted the open-source Java library named jFuzzyLogic to implement the proposed system. iFuzzyLogic is an open-source Java library available online through the proposed architecture. We ran 60 independent simulations to simulate the results we presented. The simulations were conducted on an Inter(R) Core (TM) i-5 2.3 GHz with a 10th-generation CPU and 14 GB of RAM with a Windows 10 OS. Table 4 shows the simulation configuration for the detailed system with the proposed scenario. Table 5 shows Resource configuration for the proposed methodology.

We carried out a number of simulations in the system based on the provided number of tasks to be processed by the system with an efficient and reliable content delivery framework. The results are compared in the simulation environment using the shortest job first (SJF), first in, first out (FIFO), and real-time task processing (RTP) scheduling techniques. The tasks are carried out to simulate the environment. We selected the number of functions and the number of VMs involved inside the system. The performance was measured using 12 VMs and 60 tasks from the VANET vehicular environment.

### 4.2. Performance Metrics

**Figure 10:**
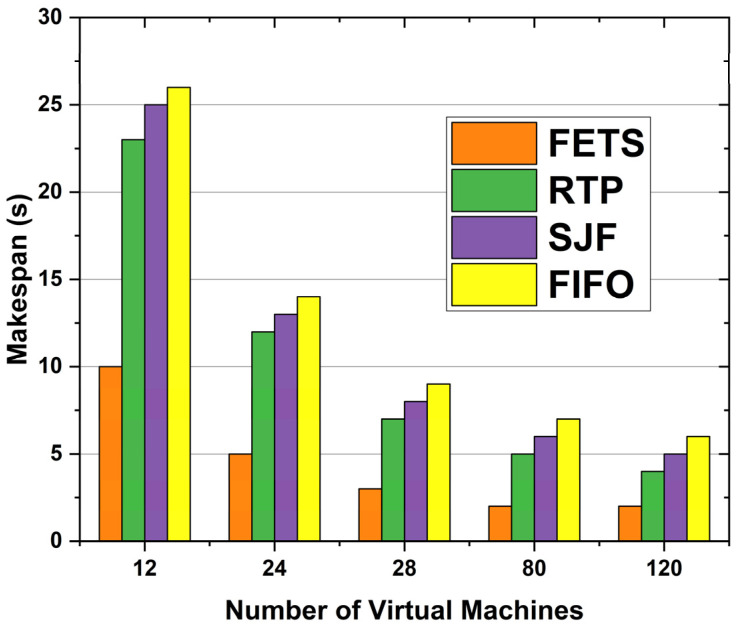
Makespan time in seconds for task offloading.

This approach uses the delay rate, average turnaround time, makespan time, success ratio, and average processing time as performance metrics. The makespan and turnaround time are computed in seconds, and the average processing time is computed in seconds. The delay rate is the delay in the task selection, which transfers the task to fog for processing. The delay is measured in seconds. The average turnaround time is the time for tasks from creation to completion of the task from fog server VMs. It is the total time spent on a task in the system. The makespan is the maximum time between when the first node to receive a task starts executing its first scheduled task and when all nodes have completed their last scheduled task. The success ratio of the tasks is the average of tasks that complete their execution from fog VMs without any interceptions. Average processing time is the time the server’s VMs take to execute the tasks. We declare the name of our approach to be fog-enabled task scheduling (FETS) in this paper’s Section 4. Figure 10 compares FETS with the first in, first out (FIFO), shortest job first (SJF), and RTP algorithms [41]. The comparison was performed using makespan time, success ratio, delay rate, and average turnaround time concerning the number of tasks and 12 VMs.

Fog-enabled task scheduling (FETS) is a methodology used in vehicular ad hoc networks (VANETs) to optimize task scheduling and resource allocation. FETS leverages the power of fog computing to perform tasks locally on a vehicle or in a nearby edge node. By doing so, it reduces the latency and bandwidth usage associated with sending data to a remote cloud server for processing. FETS also considers the mobility of vehicles in the network and dynamically adapts the task scheduling and resource allocation to account for changes in the network topology. This approach not only improves the efficiency of the network but also enhances the user experience by reducing delays and improving response times.

### 4.3. Results

When we increase the number of tasks, the time taken increases with the ratio of the number of tasks, and the delay caused stops the tasks from being executed within the specified deadline. Figure 10 shows the 120 VMs according to makespan time in seconds. When the VM increased, makespan time decreased for FETS. The proposed methodology FETS shows a shorter makespan time with higher performance. The VMs used in the proposed model are compared through different models. Our model FETS performs better from the start of task scheduling to the return of results from the fog layer (makespan time). The FETS consumes less time than the existing RTP, SJF, and FIFO techniques from task submission to task processing and returns the results to the vehicle. On 12 VMs, the proposed technique consumes 10 s makespan time. On 24 VMs, it consumes 5 s makespan time. On 28 VMs, it consumes 2.5 s makespan time. On 80 VMs, it consumes 2 s; on 120 VMs, it consumes 1.5 s makespan time in task offloading toward the fog cloud. 

**Figure 11:**
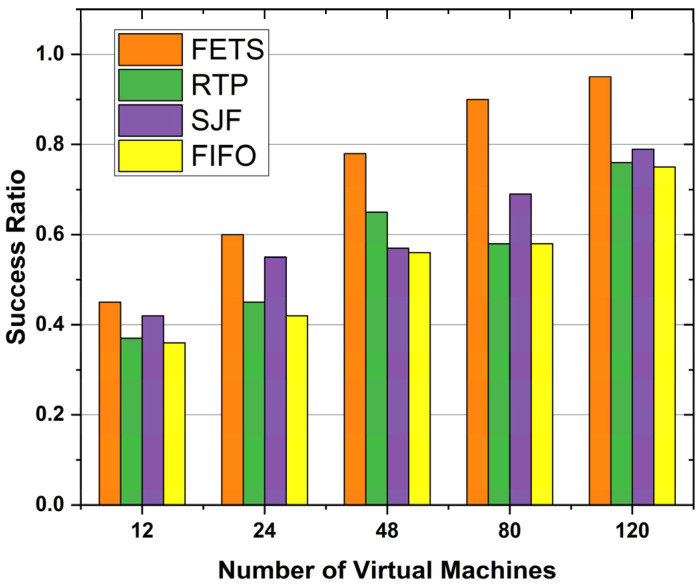
Success ratio for tasks offloading on VMs.

The success ratio shows the successful execution of the tasks using the fog VM. The number of VM allocations and success ratios is the dependent parameter for comparing the task offloading success ratio. Figure 11 shows VMs with a success ratio. The success ratio shows that our proposed technique showed a higher success ratio when the number of VMs increased to 120. The results show the consistent performance of the proposed method. The figure shows the FETS task success ratio (whole tasks submitted to fog and successful return results) based on VMs. The ratio was prepared based on the total number of tasks submitted and the total tasks returned after processing from the fog layer. The task success ratio was measured for 12, 24, 28, 80, and 120 VMs, and the success ratio is higher than those of the existing state-of-the-art FIFO, SJF, and RTP techniques. These techniques perform better compared with other techniques. The success ratio of the task migration is measured in percentages from 0 to 100. At every point, the FETS technique migrates a higher number of tasks. 

**Figure 12:**
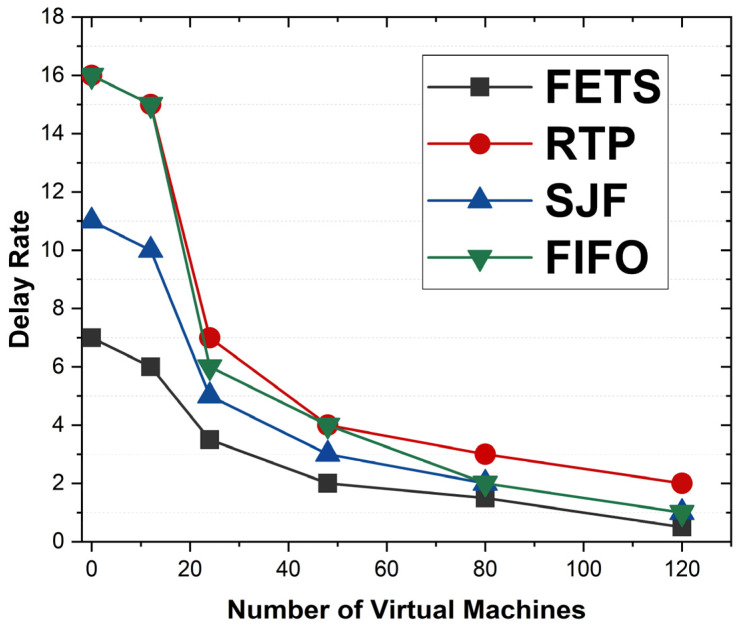
Delay in FETS, RTP, SJF, and FIFO techniques.

The delay rate is the time for offloading the tasks toward the fog VMs. The delay rate is shown in Figure 12, with the number of VMs’ performance on the horizontal axis and the delay rate on the vertical axis. The proposed FETS technique shows less delay in task submission to the fog layer. At the start, the FETS displays a low delay rate of 7. On 20 VMs, utilization shows a rate of 6 and decreases to 120 VMs. The results show that FETS has a lower delay rate when tasks are required for scheduling to the fog layer during fog broker and scheduler task processing compared with the RTP, SJF, and FIFO techniques. This technique shows the delay rate comparison. FETS shows a lower delay rate under 20, 40, 60, 80, 100, and 120 VMs. The delay rate is computed using the under-processed efficiency rules. 

**Figure 13:**
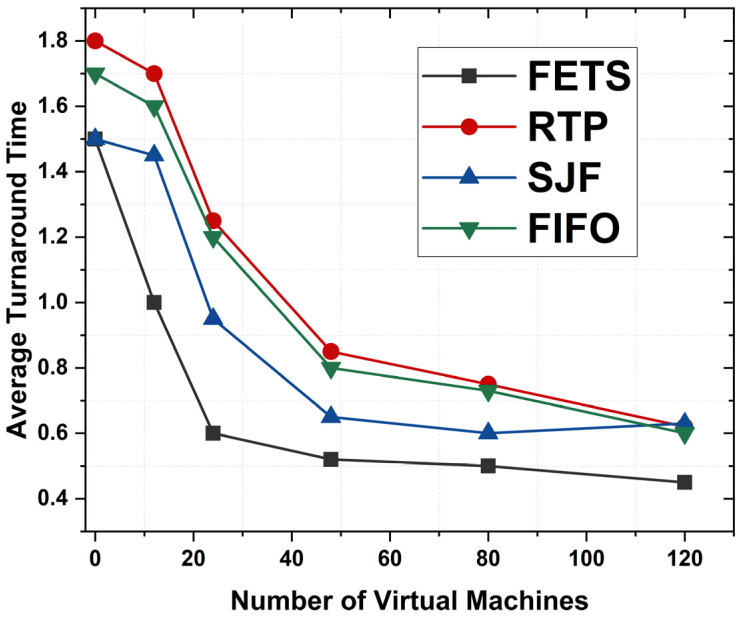
Average turnaround times between FETS, RTP, SJF, and FIFO.

The average turnaround time is the time required from task submission to completion. Figure 13 shows the average turnaround time from task submission for scheduling from vehicles and until completion from fog VMs. Figure 13 shows that FETS shows a shorter turnaround time for all tasks that are adequately migrated toward the fog layer. The lower turnaround time shows that when the VM increases, the system’s performance increases with more orders. The average turnaround time for FETS from task submission to execution is shown at less than 1 to 120 VMs. The results show the proposed FETS methodology works better for average turnaround time for task scheduling in VANET. This method computes the overall time from process submission to its completion. The FETS shows lower time consumption than the RTP, SJF, and FIFO techniques. These techniques explore the efficiency and delivery rules to maintain the relationship between the different aspects of the system. 

**Figure 14:**
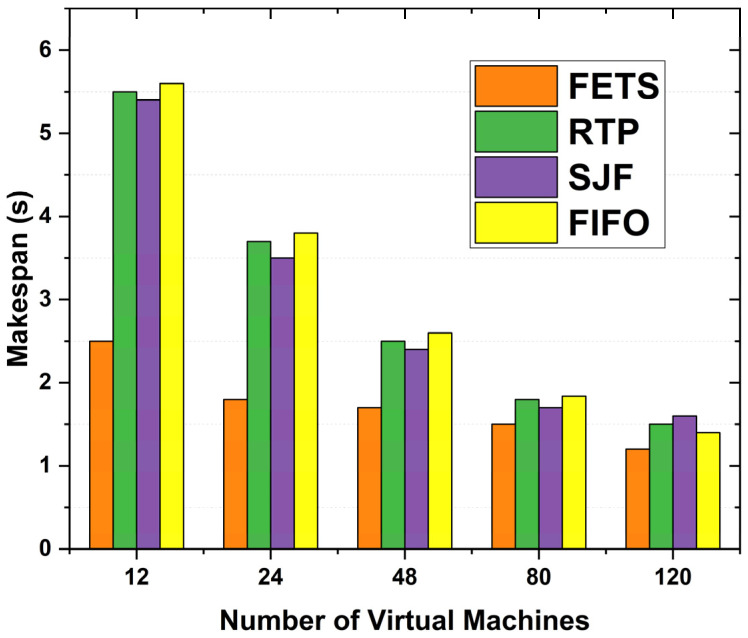
Makespan time over 60 tasks.

The fog resources are proportional to the availability of VMs at each fog layer. Makespan time elapses from the start of work to the end. The comparative benefits of the results for makespan time enhance the FETS’s scalability for the proposed techniques. Figure 14 compares makespan time over the number of VMs for the proposed FETS technique with the RTP, SJF, and FIFO techniques. The more fog VMs there are, the more resources are adopted. With the increase in VMs, the resources are synchronously enhanced. When the VMs increase, resources and available tasks are offloaded toward the fog layer. The tasks are processed using available VMs and returned to VANET for vehicle delivery. Figure 14 shows the results for 60 tasks initiated from vehicles and scheduled to fog VMs after passing from Algorithms 1 and 2. The technique effectively enhances the working experience and suggests the time to complete these VMs’ task distribution mechanism. The tasks from RTP, SJF, and FIFO are the required features for providing the listed data under the provision of the lists. The FETS method shows a lower manager for the provision of the results with a lower makespan time under the higher considerations of the performance of the proposed system. 

**Figure 15:**
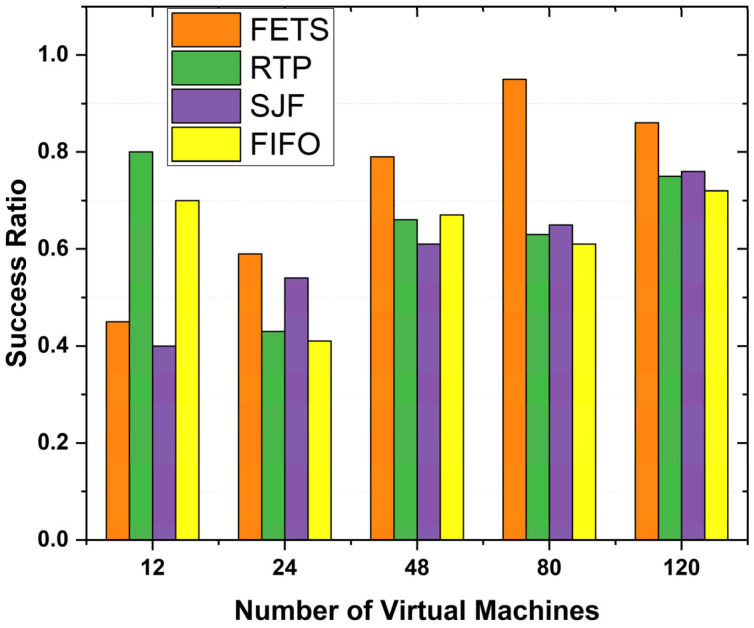
Success ratio of task migration over 60 tasks with VMs.

Therefore, in Figure 15, we observe the decrease in makespan time, average turnaround time, and delay rate of the tasks to be performed. The proposed technique’s success ratio is 60 vehicle tasks with 120 VMs. The proposed technique, FETS, is compared with RTP, SJF, and FIFO with 120 VMs for a successful ratio of task execution completion using the fog. The number of tasks in the successful execution ratio of the proposed FETS technique shows that a higher number of tasks obtain success and there is a lower failure ratio compared with RTP, SJF, and FIFO. The success ratio is the percentage of the total submitted and successfully executed tasks. The success ratio is the total information that can demonstrate the actual performance comparison of the FETS method with the state-of-the-art existing RTP, SJF, and FIFO techniques. Under 12 VMs, the success ratio of the proposed technique drops, but under 24, 48, 80, and 120 VMs, it is increased toward those of the other techniques. There are several resources with the ability to demonstrate this knowledge. 

**Figure 16:**
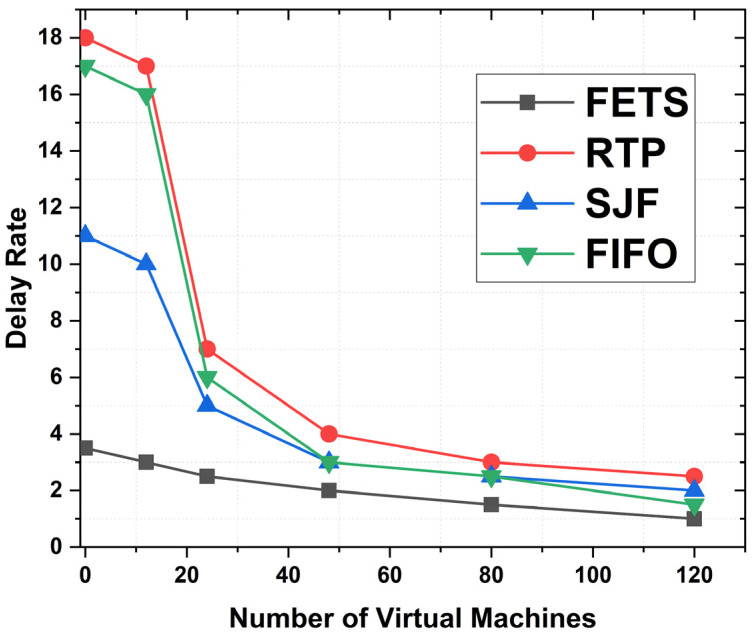
Delay rate of tasks with VM migration for 120 tasks.

The delay rate is the delay observed during the task scheduling and offloading to the fog VM. The delay rate is taken from makespan. The comparison between the RTP, SJF, and FIFO techniques and our proposed technique, FETS, is observed. In Figure 16, the delay in task execution is observed. The proposed FETS algorithm executes the tasks based on the availability of resources like bandwidth, memory, storage, power, and CPU utilization. The delay rate of FETS is of less concern than those of other techniques, from 1 to 120 VMs over 120 tasks. The delay rate is lower than those of the RTP, SJF, and FIFO techniques with 20, 40, 60, 80, 100, and 120 VMs. The delay rate shows better performance for the FETS than the RTP, SJF, and FIFO techniques in enhancing the proposed solution and elaborating the performance metrics effectively. There are several techniques proposed with abilities to demonstrate. 

**Figure 17:**
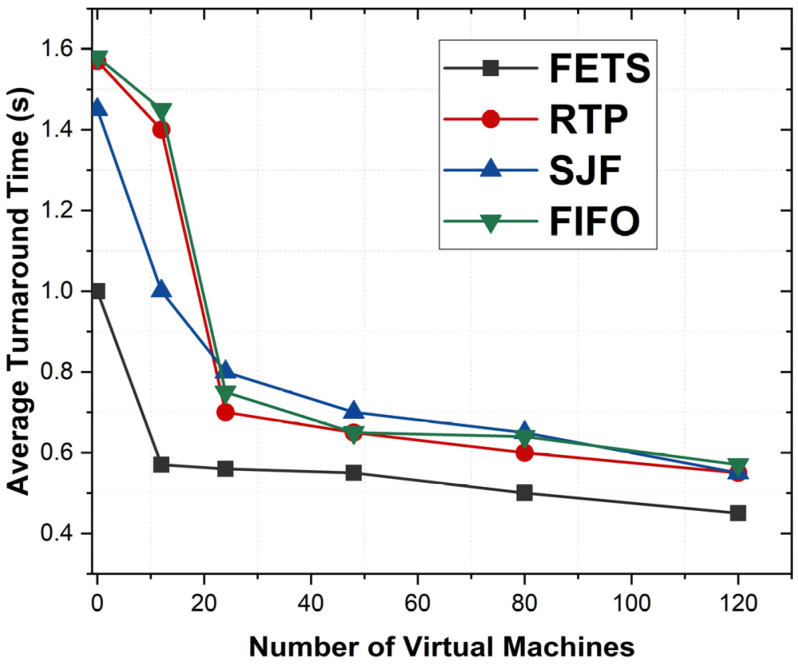
Average turnaround time for each task for VMs for 120 tasks.

The proposed FETS algorithm takes the task deadline as one of the essential parameters for the performance evaluation, such as delay rate, makespan time, turnaround time, and average turnaround time. The deadline is fetched from tasks initiated by vehicles and forwarded to the fog/cloud. Algorithm 2 forwards the vehicle’s tasks toward fog VMs to process after the fuzzy logic decision. Figure 17 shows the average turnaround time over the number of VMs deputed to handle the 120 tasks. Figure 17 shows that the proposed FETS method shows less turnaround time overall for 120 tasks submitted to fog VMs. The proposed technique executes the maximum number of tasks. The tasks are forwarded with better efficiency, power, CPU, bandwidth, storage, and memory requirements for the task selection and processing for fuzzy logic decision-making. The graph shows the consistent processing power and time constraints for logical decision-making. The average time is computed in seconds from 20 to 120 VMs. FETS shows a higher level of demand than the existing methodologies for performance measurements. Several provisions of the results make the system more vulnerable and enhance the working criteria of these tasks. 

**Figure 18:**
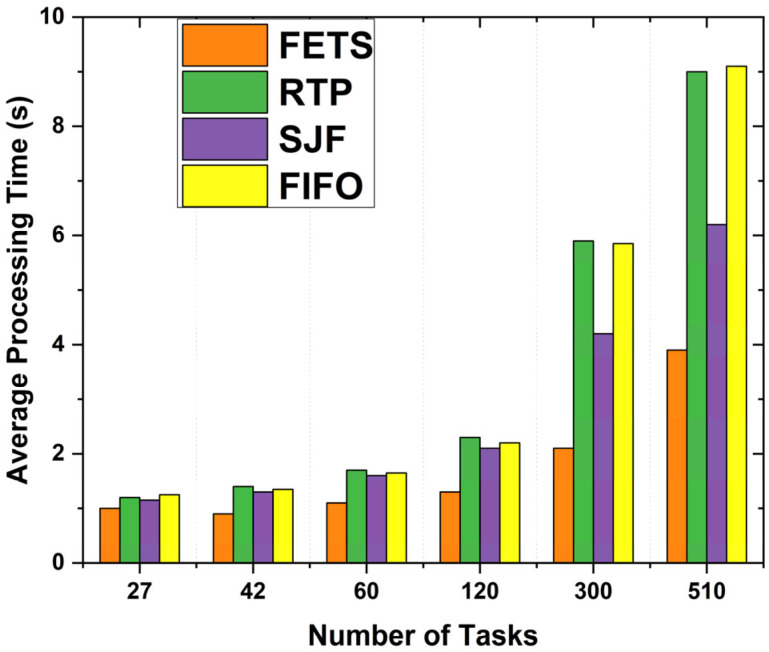
Processing time with VMs.

The processing time over VMs is when the task is submitted to the VM and that VM processes the task and provides the results back to the scheduler. We take the number of tasks on the horizontal axis and compare it with the average processing time in seconds on the vertical axis to compare the results of the proposed FETS method with RTP, SJF, and FIFO. Using this methodology, these are the numbers of tasks with less transmission time. Under such a situation, the proposed FETS methodology assigns the tasks to VMs in a reasonable amount of time. Figure 18 shows that when there is an increase in the number of tasks to 300 or 510, the proposed FETS technique performs better than other approaches. It effectively reduces the waiting time for task assignments under the required resources. The proposed FETS technique performs better than the RTP, SJF, and FIFO techniques. The results indicate that average processing time enhances the average processing time for task submission and completion.

**Figure 19:**
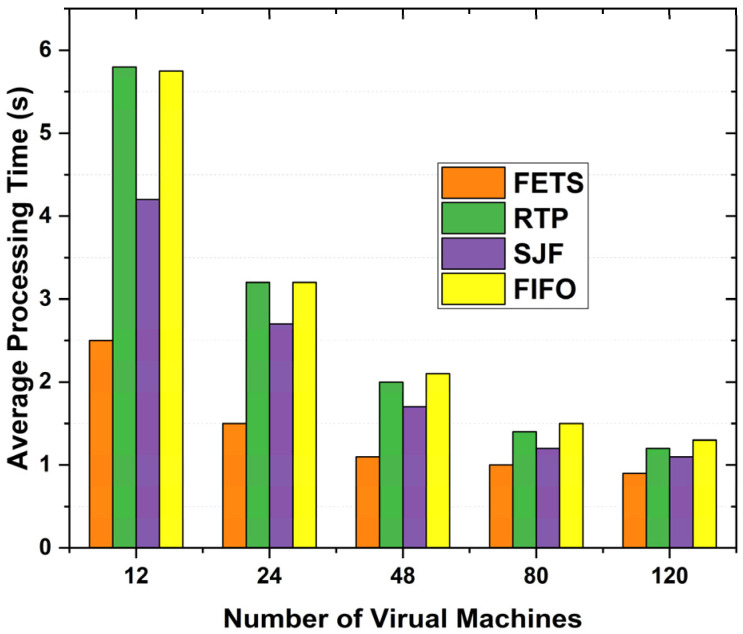
Average processing time with 60 tasks.

The average processing time for tasks shows the time from task submission to completion. When the average processing time decreases, the capabilities of VMs become higher. Figure 19 shows the proposed technique used on 60 tasks assigned toward fog computing. The tasks are assigned to the least loaded VM of fog from vehicles, showing the effective system performance. Thus, this reduces the system’s waiting time for further reduction in the long-term processing of the assigned tasks.

Moreover, we observed that the FETS algorithm works in less processing time and performs better than the RTP, SJF, and FIFO algorithms under the proposed parameters: average processing time with the ability to demonstrate the working performance of the proposed techniques. The average time is computed with 60 tasks for 120 VMs. These tasks are computed, and average processing times are calculated. FETS shows better results with a shorter average processing time, making the system faster than existing techniques. 

**Figure 20:**
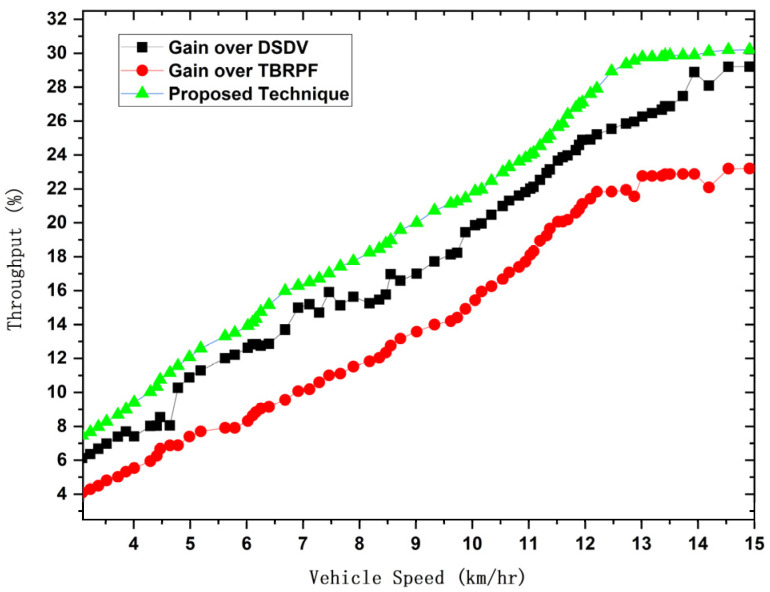
Network throughput for the suggested method compared with previous findings.

In this network, we carefully check flow by looking at where vehicles start and how they interact with intensity levels set to match vehicle speeds. This link is shown visually in Figure 20. It is important to note that figuring out packet drops is an important part of this evaluation because it requires following the gearbox routes in different car communication situations. Throughput is the synchronization of large data bits, including their sending and receiving at different speeds, to get a full picture of how well a network works. We compare the proposed method’s throughput with well-known protocols such as the destination-sequenced distance-vector routing protocol (DSDV) and the temporally ordered routing algorithm for mobility (TORA), which is based on the reverse-path forwarding protocol (TBRFP). DSDV is a good choice. After all, it works well in static networks because it is proactive and keeps routes stable. Previous studies used DSDV often when route updates had to happen all the time, like in sensor networks. Based on TBRFP, TORA was chosen because it can work in changing settings and takes a flexible approach. Previous research has successfully used TORA in mobile ad hoc networks, showing that it can handle frequent network structure changes. These routing protocols can handle different situations. DSDV works best in static setups, while TORA, based on TBRFP, handles the problems with mobile and changing network settings. The suggested method’s throughput is compared with that of DSDV and TORA. The results show that the proposed method has a higher network throughput and is more efficient at routing for better performance.

### 4.4. Discussion

The presented paper focuses on evaluating the performance of the proposed fog-enabled task scheduling (FETS) methodology in vehicular ad hoc networks (VANETs) using a simulation environment. The simulation was conducted using iFogSim, a technology built on CloudSim and coded in Java, with 60 independent simulations executed on a specified hardware configuration. The proposed methodology is compared against existing scheduling techniques, including shortest job first (SJF), first in, first out (FIFO), and real-time task processing (RTP). The performance metrics utilized for evaluation include delay rate, average turnaround time, makespan time, success ratio, and average processing time. The simulation results reveal that FETS outperforms RTP, SJF, and FIFO across these metrics. Notably, FETS demonstrates a decrease in makespan time as the number of virtual machines (VMs) increases, indicating scalability and efficiency in task offloading.

The success ratio in FETS increases as the number of VMs increases, highlighting its effectiveness in task execution and completion. The delay rate in FETS is consistently lower than those in RTP, SJF, and FIFO, emphasizing its efficiency in task submission to the fog layer. Average turnaround time and processing time also exhibit favorable results for FETS, showcasing its ability to enhance system performance by reducing task completion times. The proposed methodology’s throughput is compared with well-known routing protocols, namely the destination-sequenced distance-vector routing protocol (DSDV) and temporally ordered routing algorithm for mobility (TORA). The results indicate that FETS achieves higher network throughput, demonstrating its efficiency in routing and overall network performance.

## 5. Conclusions with Future Directions

### 5.1. Conclusions

This research presents a novel fuzzy logic-based task scheduling technique in VANETs. The proposed FETS model and simulation results show that the proposed technique intelligently decides and distributes the tasks toward the fog layer for VM computations. This research also uses the cloud server to distribute the tasks toward the cloud enhancements. The task distribution on the fog VMs is considered based on the resource-handling capacity of fog VMs over the number of assigned tasks. The scheduling algorithm performs better for task management and scheduling. Based on the simulation results, the tasks distributed to fog layers are effectively utilized and processed using the proposed FETS technique. The results in Section 4 of this paper were compared with existing techniques, and experiments were performed. The delay rate, average turnaround time, and makespan time were considered for task processing and results enhancement. Lower resources and VMs are utilized in the proposed technique during task processing and scheduling toward the fog or cloud. The waiting time for the tasks is also reduced due to the proposed technique, with efficient results and the maintained or proposed algorithm. 

### 5.2. Future Research Plan

The proposed future research plan for fog-enabled task scheduling (FETS) for energy consumption and performance measures will involve a comprehensive investigation of the existing scheduling algorithms used in fog computing, their limitations, and their impact on energy consumption and performance. The research will develop novel scheduling algorithms considering energy consumption and performance measures as primary optimization metrics. These algorithms will be evaluated through extensive simulations and experimental studies in a realistic fog computing environment. Additionally, the research will investigate the potential impact of different factors, such as workload characteristics, device heterogeneity, and network topology, on the performance and energy consumption of the proposed algorithms. Finally, the research will develop best practices and guidelines for deploying fog computing-based applications that effectively balance energy consumption and performance measures.

FETS has the potential to be widely adopted in a variety of industries, such as smart cities, smart homes, industrial IoT, and transportation, where reducing energy consumption and optimizing performance are essential. However, further research is needed to fully realize the potential of FETS and address some challenges, such as security and privacy, task prioritization, and resource allocation.

Limitations of the proposed approach are as follows:

- The research is limited to adopting fuzzy logic techniques only.
- Fog computing may provide limited computational abilities near the vehicular devices, and to handle data-intensive tasks, cloud layer adoption is required.

## Figures and Tables

**Table 1:** The fuzzy sets with input and output parameter values.

Author and Year	Problem	Methodology	Contributions	Limitations
Gupta et al. (2018) [34]	Limited task scheduling efficiency in IoV	Machine learning	Proposes a novel fuzzy logic-based approach for efficient task scheduling in IoV, enhancing overall system performance.	Limited to simulation-based validation; real-world applicability requires exploration.
Chen and Wang (2017) [35]	Challenges in task offloading in fog computing	Optimization algorithms	Investigates task offloading challenges in fog computing, providing insights into efficient resource utilization.	It focuses on general fog computing; specific IoV considerations need further exploration.
Zhang et al. (2019) [36]	Bandwidth constraints in VANET	Genetic algorithms	Addresses bandwidth limitations in VANET, offering a genetic algorithm-based solution for improved data transmission.	Lacks consideration for the broader IoV ecosystem and fog-based processing.
Lee and Kim (2020) [37]	Latency issues in IoV	Deep learning	Examines latency issues in IoV, introducing a deep learning approach to minimize response times.	Limited discussion of task scheduling; focuses more on latency mitigation.
Wang et al. (2016) [38]	Resource constraints in fog computing	Heuristic approaches	Investigates resource constraints in Fog computing, proposing heuristic methods for optimal resource allocation.	Limited emphasis on fuzzy logic does not explicitly address IoV-based task scheduling.
Liu et al. (2018) [39]	Vehicle mobility challenges in IoV	Mathematical modeling	Explores challenges related to vehicle mobility in IoV, leveraging mathematical models for predictive analysis.	Limited discussion of task scheduling methodologies; focuses more on mobility modeling.
Sharma and Patel (2021) [40]	Inefficiencies in VANET processing power	Metaheuristic algorithms	Introduces metaheuristic algorithms to optimize processing power usage in VANETs.	Lacks exploration of fuzzy logic-based approaches and broader IoV context.
Proposed Methodology	VANET faces task scheduling challenges	Fuzzy logic-based RSU-less IoV scheduling approach	Our contribution involves introducing a fuzzy logic-based fog-enabled task scheduling technique in VANETs to enhance task time and energy efficiency. Leveraging fog and fuzzy logic, our model efficiently schedules resource-intensive tasks evaluated using Mumdani fuzzification. We designed a V2V-based fuzzy logic VANET architecture that demonstrates superior simulation performance.	Lack of implementation of machine learning techniques that can more effectively schedule the tasks.

**Table 2:** The fuzzy sets with input and output parameter values.

CPU	Storage	Bandwidth	Latency	Task_Deadline	Task_Execution
Low	Low	Low	Low	Low	Cloud_Layer
Low	Low	Low	Low	Low	Fog_Layer
Low	Low	Low	Low	Medium	Cloud_Layer
Low	Low	Low	Low	Medium	Fog_Layer
Low	Low	Low	Low	High	Cloud_Layer
Low	Low	Low	Low	High	Fog_Layer
Low	Low	Low	Medium	Low	Cloud_Layer
Low	Low	Low	Medium	Low	Fog_Layer
Low	Low	Low	Medium	Medium	Cloud_Layer
Low	Low	Low	Medium	Medium	Fog_Layer
Low	Low	Low	Medium	High	Cloud_Layer
Low	Low	Low	Medium	High	Fog_Layer
Low	Low	Low	High	Low	Cloud_Layer
Low	Low	Low	High	Low	Fog_Layer
Low	Low	Low	High	Medium	Cloud_Layer
Low	Low	Low	High	Medium	Fog_Layer
Low	Low	Low	High	High	Cloud_Layer
Low	Low	Low	High	High	Fog_Layer
Low	Low	Medium	Low	Low	Cloud_Layer
Low	Low	Medium	Low	Low	Fog_Layer
Low	Low	Medium	Low	Medium	Cloud_Layer
Low	Low	Medium	Low	Medium	Fog_Layer
Low	Low	Medium	Low	High	Cloud_Layer
Low	Low	Medium	Low	High	Fog_Layer
Low	Low	Medium	Medium	Low	Cloud_Layer
Low	Low	Medium	Medium	Low	Fog_Layer
Low	Low	Medium	Medium	Medium	Cloud_Layer
Low	Low	Medium	Medium	Medium	Fog_Layer
Low	Low	Medium	Medium	High	Cloud_Layer
Low	Low	Medium	Medium	High	Fog_Layer
Low	Low	Medium	High	Low	Cloud_Layer
Low	Low	Medium	High	Low	Fog_Layer
Low	Low	Medium	High	Medium	Cloud_Layer
Low	Low	Medium	High	Medium	Fog_Layer
Low	Low	Medium	High	High	Cloud_Layer
Low	Low	Medium	High	High	Fog_Layer
Low	Low	High	Low	Low	Cloud_Layer
Low	Low	High	Low	Low	Fog_Layer
Low	Low	High	Low	Medium	Cloud_Layer
Low	Low	High	Low	Medium	Fog_Layer
Low	Low	High	Low	High	Cloud_Layer
Low	Low	High	Low	High	Fog_Layer
Low	Low	High	Medium	Low	Cloud_Layer
Low	Low	High	Medium	Low	Fog_Layer
Low	Low	High	Medium	Medium	Cloud_Layer
Low	Low	High	Medium	Medium	Fog_Layer
Low	Low	High	Medium	High	Cloud_Layer
Low	Low	High	Medium	High	Fog_Layer
Low	Low	High	High	Low	Cloud_Layer
Low	Low	High	High	Low	Fog_Layer
Low	Low	High	High	Medium	Cloud_Layer
Low	Low	High	High	Medium	Fog_Layer
Low	Low	High	High	High	Cloud_Layer
Low	Low	High	High	High	Fog_Layer
Low	Medium	Low	Low	Low	Cloud_Layer
Low	Medium	Low	Low	Low	Fog_Layer
Low	Medium	Low	Low	Medium	Cloud_Layer
Low	Medium	Low	Low	Medium	Fog_Layer
Low	Medium	Low	Low	High	Cloud_Layer
Low	Medium	Low	Low	High	Fog_Layer
Low	Medium	Low	Medium	Low	Cloud_Layer
Low	Medium	Low	Medium	Low	Fog_Layer
Low	Medium	Low	Medium	Medium	Cloud_Layer
Low	Medium	Low	Medium	Medium	Fog_Layer
Low	Medium	Low	Medium	High	Cloud_Layer
Low	Medium	Low	Medium	High	Fog_Layer
Low	Medium	Low	High	Low	Cloud_Layer
Low	Medium	Low	High	Low	Fog_Layer
Low	Medium	Low	High	Medium	Cloud_Layer
Low	Medium	Low	High	Medium	Fog_Layer
Low	Medium	Low	High	High	Cloud_Layer
Low	Medium	Low	High	High	Fog_Layer
Low	Medium	Medium	Low	Low	Cloud_Layer
Low	Medium	Medium	Low	Low	Fog_Layer
Low	Medium	Medium	Low	Medium	Cloud_Layer
Low	Medium	Medium	Low	Medium	Fog_Layer
Low	Medium	Medium	Low	High	Cloud_Layer
Low	Medium	Medium	Low	High	Fog_Layer
Low	Medium	Medium	Medium	Low	Cloud_Layer
Low	Medium	Medium	Medium	Low	Fog_Layer
Low	Medium	Medium	Medium	Medium	Cloud_Layer
Low	Medium	Medium	Medium	Medium	Fog_Layer
Low	Medium	Medium	Medium	High	Cloud_Layer
Low	Medium	Medium	Medium	High	Fog_Layer
Low	Medium	Medium	High	Low	Cloud_Layer
Low	Medium	Medium	High	Low	Fog_Layer
Low	Medium	Medium	High	Medium	Cloud_Layer
Low	Medium	Medium	High	Medium	Fog_Layer
Low	Medium	Medium	High	High	Cloud_Layer
Low	Medium	Medium	High	High	Fog_Layer
Low	Medium	High	Low	Low	Cloud_Layer
Low	Medium	High	Low	Low	Fog_Layer
Low	Medium	High	Low	Medium	Cloud_Layer
Low	Medium	High	Low	Medium	Fog_Layer
Low	Medium	High	Low	High	Cloud_Layer
Low	Medium	High	Low	High	Fog_Layer
Low	Medium	High	Medium	Low	Cloud_Layer
Low	Medium	High	Medium	Low	Fog_Layer
Low	Medium	High	Medium	Medium	Cloud_Layer
Low	Medium	High	Medium	Medium	Fog_Layer
Low	Medium	High	Medium	High	Cloud_Layer
Low	Medium	High	Medium	High	Fog_Layer
Low	Medium	High	High	Low	Cloud_Layer
Low	Medium	High	High	Low	Fog_Layer
Low	Medium	High	High	Medium	Cloud_Layer
Low	Medium	High	High	Medium	Fog_Layer
Low	Medium	High	High	High	Cloud_Layer
Low	Medium	High	High	High	Fog_Layer
Low	High	Low	Low	Low	Cloud_Layer
Low	High	Low	Low	Low	Fog_Layer
Low	High	Low	Low	Medium	Cloud_Layer
Low	High	Low	Low	Medium	Fog_Layer
Low	High	Low	Low	High	Cloud_Layer
Low	High	Low	Low	High	Fog_Layer
Low	High	Low	Medium	Low	Cloud_Layer
Low	High	Low	Medium	Low	Fog_Layer
Low	High	Low	Medium	Medium	Cloud_Layer
Low	High	Low	Medium	Medium	Fog_Layer
Low	High	Low	Medium	High	Cloud_Layer
Low	High	Low	Medium	High	Fog_Layer
Low	High	Low	High	Low	Cloud_Layer
Low	High	Low	High	Low	Fog_Layer
Low	High	Low	High	Medium	Cloud_Layer
Low	High	Low	High	Medium	Fog_Layer
Low	High	Low	High	High	Cloud_Layer
Low	High	Low	High	High	Fog_Layer
Low	High	Medium	Low	Low	Cloud_Layer
Low	High	Medium	Low	Low	Fog_Layer
Low	High	Medium	Low	Medium	Cloud_Layer
Low	High	Medium	Low	Medium	Fog_Layer
Low	High	Medium	Low	High	Cloud_Layer
Low	High	Medium	Low	High	Fog_Layer
Low	High	Medium	Medium	Low	Cloud_Layer
Low	High	Medium	Medium	Low	Fog_Layer
Low	High	Medium	Medium	Medium	Cloud_Layer
Low	High	Medium	Medium	Medium	Fog_Layer
Low	High	Medium	Medium	High	Cloud_Layer

**Table 3:** Values of the respective parameters alongside the fuzzy rules.

Input/Output Variables	Fuzzy Sets
Task execution	Cloud layer, fog layer
Latency	Low, medium, and high
Deadline of task	Low, medium, and high
Storage	Low, medium, and high
CPU	Low, medium, and high
Bandwidth	Low, medium, and high

**Table 4:** Notations with descriptions are used in the algorithms and equations.

S.No.	Notation	Description
1	VM	Virtual machines are used in fog computing to process the scheduled task.
2	Bandwidth	Utilized bandwidth of the proposed system
3	Task Deadline	The total time each task is processed on the fog VM
4	$packer_{id}$	An ID is associated with each of the tasks.
5	Node	The vehicular node
6	GPS Coordinates	Obtain total GPS coordinates for the final version of the system.
7	Hop count $n_{i}$	The number of intermediate nodes passed from the system to another system
8	$id_{i}$	The ID associated with each identifier
9	Lower_limit()	The lower limit threshold is set for the waiting time of the scheduled task.
10	Min−waiting−time	Time is required to process the task toward the final delivered values.
11	Drop()	Drop the packet before schedule because the threshold limit is exceeded.
12	V	Set of all VMs in the fog layer of the proposed model
13	$V_{id}$	Unique identifier for the VM
14	T	Total time for task submission toward processing
15	NL	Network latency for the proposed system
16	Q	Bandwidth associated with fog layer
17	FN	Fog network
18	fog_cloud()	Fog cloud-based identifier
19	FLDA	Fuzzy logic-based decision algorithm
20	dl	Decision logic
21	$Z_{T}$	Scheduled task uploaded toward the fog cloud

**Table 5:** Resource configuration for the proposed methodology.

Process Type	CPU (Mips)	Storage (Gigabyte)	Memory (Megabyte)	Bandwidth Mbps
Cloud	42,700	1,000,000	40,000	10,000
Fog	20,600	10,000	10,000	100,000
VM (Computational)	1400	1000	1500	1000
VM (Storage)	900	1500	1000	1000
VM (Bandwidth)	1000	1000	1000	1500
VM (Standard)	1500	1500	1500	1500

## Data Availability

The data presented in this study are available upon reasonable request from the corresponding author.
